# Validation of the X-ray microtomography in the assessment of duodenal morphometry and surface area in celiac disease

**DOI:** 10.3389/fimmu.2022.945197

**Published:** 2022-09-23

**Authors:** Johannes Virta, Markus Hannula, Katri Lindfors, Ilmari Tamminen, Juha Taavela, Heini Huhtala, Katri Kaukinen, Päivi Saavalainen, Jari Hyttinen, Kalle Kurppa

**Affiliations:** ^1^ Tampere Center for Child Health Research, Faculty of Medicine and Health Technology, Tampere University, Tampere, Finland; ^2^ Department of Pediatrics, Tampere University Hospital, Tampere, Finland; ^3^ Computational Biophysics and Imaging Group, The Faculty of Medicine and Health Technology, Tampere University, Tampere, Finland; ^4^ Celiac Disease Research Center, Faculty of Medicine and Health Technology, Tampere University, Tampere, Finland; ^5^ Faculty of Social Sciences, Tampere University, Tampere, Finland; ^6^ Department of Internal Medicine, Tampere University Hospital, Tampere, Finland; ^7^ Department of Medical and Clinical Genetics, University of Helsinki, Helsinki, Finland; ^8^ The University Consortium of Seinäjoki and Seinäjoki Central Hospital, Seinäjoki, Finland

**Keywords:** celiac disease, biopsies, histology, diagnosis, micro-CT, imaging

## Abstract

**Background:**

Duodenal histology remains the diagnostic reference standard in celiac disease. However, traditional methods have suboptimal sensitivity and reproducibility for early mucosal changes and research purposes. We validated a recently introduced micro-CT imaging method for an accurate digital evaluation of duodenal histomorphometry and mucosal surface areas.

**Methods:**

Endoscopic biopsies from 58 individuals were utilized for the micro-CT imaging, selecting histological changes ranging from normal to severely damaged mucosa. The imaging protocol was optimized for practicability and resolution. The Bland–Altman method was applied to test intra- and interobserver variations in the blinded measurements.

**Results:**

The 3D micro-CT reconstructions enabled easy and precise digital cutting with optimal orientation and computer-assisted measurement of the surface area. Intraobserver analysis of morphological measurements showed a mean difference of 0.011 with limits of agreement (LA) from -0.397 to 0.375 and a standard deviation (SD) of 0.197. The corresponding figures for interobserver analysis were 0.080, from -0.719 to 0.537 and 0.320, respectively. The intraclass correlation coefficients (ICC) for the intraobserver and interobserver variations were 0.981 and 0.954, respectively. Intraobserver surface area analysis yielded a mean difference of 0.010, LA from -0.764 to 0.785 and an SD of 0.395, and an interobserver analysis mean difference of 0.028, LA from -0.642 to 0.698 and SD of 0.342. The respective ICCs for the intra- and interobserver variations were 0.963 and 0.972.

**Conclusions:**

Micro-CT showed excellent accuracy and reproducibility in the evaluation of mucosal morphometry and surface areas. The improved sensitivity for histological changes is a powerful tool for the diagnosis of celiac disease and for clinical and pharmacological studies.

## Introduction

Celiac disease (CeD) is an immune-mediated gastrointestinal condition with an estimated prevalence of 1%–2% (1). Although the role of serological tests and other surrogate markers for tissue damage in CeD is increasing (2, 3), duodenal histopathology remains the reference standard for the diagnosis and evaluation of treatment response, as well as for the emerging pharmaceutical trials (4, 5). Histological assessment, however, is complicated by the variable quality of the endoscopic specimens and patchiness of the mucosal lesion (6). Moreover, precise orientation of the biopsy cuttings needed for reliable measurements is problematic and often not achieved (7, 8). These challenges have led to significant intra- and interobserver variation in the interpretation of histology, emphasizing the need for more accurate morphometric readouts (3, 4, 6, 8–13).

X-ray microtomography (micro-CT) is an imaging technique enabling comprehensive virtual modeling of a tissue sample with high resolution and with staining methods, also for soft tissue (14, 15). The resulting 3D reconstructions can be freely rotated and digitally cut and quantified, making the method particularly promising for morphometric measurements. We recently optimized a micro-CT protocol for human-derived intestinal biopsies, and, according to the preliminary results, the method provides superior accuracy compared with traditional histology (16). Moreover, we were able to measure mucosal surface areas from the 3D reconstructions for biologically more informative and more sensitive readouts. These findings suggest that micro-CT is a promising tool for the assessment of duodenal mucosa, but further validation is required before widespread clinical use.

We here proceed to further study and validate the diagnostic accuracy of the micro-CT imaging by utilizing small-bowel mucosal samples representing variable degrees of histological changes taken from a large cohort of CeD patients and controls.

## Materials and methods

### Patient and study design

The study was carried out at Tampere University and Tampere University Hospital. Duodenal biopsies for micro-CT imaging were chosen from 58 individuals who had undergone esophagogastroduodenoscopy for the diagnosis or follow-up of CeD or due to other clinical indication and had given permission for the samples to be used for research purposes. The aim was to collect histologic changes of variable degrees, ranging from morphologically normal intestinal villi to completely flat duodenal mucosa, thus representing both non-CeD patients and different stages of disease activity. Besides the endoscopy, CeD-associated serology and HLA genetics were also measured. Subjects having received a CeD diagnosis started on a strict gluten-free diet after guidance by a dietician, and a control visit including a repeat biopsy was scheduled approximately after 1 year. The biopsies obtained were used for the study analyses as described below.

The Regional Ethics Committee of Pirkanmaa Hospital District approved the original patient recruitment. Written informed consent was requested from all subjects participating in the research projects, and the Declaration of Helsinki was adhered to.

### Serology and genetics

Serum IgA-class tissue transglutaminase antibodies (TGA) were measured by commercial enzyme-linked immunosorbent assay (Phadia AB, Uppsala, Sweden), considering values ≥5.0 U/ml positive. Serum endomysium antibodies (EmA) were measured by an in-house immunofluorescence method as described elsewhere in detail (17). A titer 1:≥5 for EmA was considered positive and further diluted up to 1:4,000. Gene alleles encoding the CeD-associated HLA-DQ2 and HLA-DQ8 haplogenotypes were analyzed using either the SSPTM DQB1 kit (Olerup SSP AB, Saltsjöbaden, Sweden) or the tag SNP method (18). Lack of these haplogenotypes has a high negative predictive value for the presence of CeD (1).

### Histology

**Figure 1:**
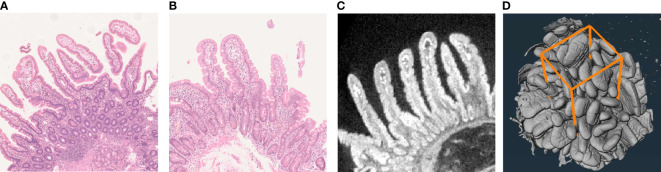
Examples of the traditional H&E-stained duodenal biopsy cuttings **(A, B)** and the corresponding micro-CT cutting **(C)** obtained from digital 3D reconstruction of up to 1,600 separate CT images **(D)**. Panel **A** demonstrates poor sample orientation, visualized by circular cross sections of the mucosal crypts and discontinuous villi, which makes accurate morphometric measurements impossible. Sections like these should not be used in the histological diagnostics of celiac disease or as an outcome measurement, e.g., in drug trials. The orientated sample in Panel **B** with longitudinally cut crypts enables a more reliable measurement. However, obtaining such a section can be laborious and time-consuming, as several recuttings and reevaluations are often needed. Moreover, even this section remains suboptimal, causing inaccuracy particularly with borderline diagnostic cases and when measuring subtle treatment responses. The corresponding digital micro-CT cutting enables easy and precise selection of the best possible section for accurate morphometry **(C)**. Quantifying the mucosal surface area **(D)** further improves measurement accuracy and reproducibility and also better reflects the actual biological phenomenon.

At least four forceps mucosal biopsies were taken from the second or third part of the duodenum for routine diagnostics using a standard endoscope. Several additional samples were taken for research purposes. For histology, the biopsies were fixed with formalin, embedded in paraffin, cut into 2-µm slices, and stained with hematoxylin–eosin (H&E). In addition to conventional grouped classification, the mucosal specimens were assessed with quantitative histomorphometry applying our validated standard operating procedures (9). Only biopsy sections with longitudinally cut villi–crypt pairs were accepted for the measurement of histomorphometry and villus height/crypt depth ratio (VH : CrD), and recuttings were made until an acceptable orientation was obtained (**Figures 1A, B** **)**. VH : CrD is reported as an average of three distinct crypt–villous pairs (9). CeD diagnosis was based on the determination of crypt hyperplasia and villous atrophy (VH : CrD <2.0) in the routine histological assessment by a hospital pathologist. Potential CeD was defined as seropositivity to TGA and/or EMA and presence of HLA-DQ2/DQ8 with non-diagnostic duodenal histology in the aforesaid histopathological evaluation (19).

### Micro-CT imaging

Paraffin-embedded duodenal samples were used for micro-CT. Excess paraffin around the actual tissue was removed from the biopsies before imaging, and the remaining sample was placed in an iodine–ethanol solution (I_2_E) for 12 h to enhance the intrinsically low soft tissue contrast (16). The I_2_E solution was made by dissolving solid iodine (207772, Sigma-Aldrich, MO) in absolute ethanol to achieve a concentration of 10 mg/ml. Although tissue saturation could theoretically be faster in fresh biopsies, according to our proof-of-concept study the paraffin-embedded samples showed fewer sample-movement artifacts with sufficient saturation (16). The contrast-enhanced samples were positioned in a 1-ml plastic syringe between two rubber pistons for mechanical stabilization, and the syringe was filled with the I_2_E to prevent outward diffusion of the contrast agent. A set of images (drift file) was collected from a fixed angle during the imaging process in order to monitor chemical and mechanical stability.

The imaging was performed by the commercial MicroXCT-400 device (Xradia, Carl Zeiss AG, CA, USA) applying an acceleration voltage of 100 kV and a 10-W source power without filtering (16). The settings used provide adequate image quality with a practicable imaging time, and no beam hardening was observed. A total of 1,600 separate X-ray projections were obtained uniformly 360° around each sample with a 5-s exposure time for each image. An X-ray detection scintillator with 10× objective was used with binning 2, delivering a voxel size of approximately 2 μm. For 3D image creation, the data were reconstructed by XMReconstructor 8.1.6599 software (Xradia). The 3D reconstructions obtained could be freely rotated and digitally cut into slices, always with optimal cutting angles for exact morphometric measurements of the villi and crypts (**Figure 1C**).

### Surface area measurements with micro-CT

The original protocol has been described elsewhere (16). Some modifications were made to enable a more efficient workflow. Briefly, the first part of the analysis was done utilizing Avizo 2020.2 software (Thermo Fisher Scientific, Waltham, MA, USA). First, a rectangular cuboid was selected from the imaging data. The volume has side lengths of 0.5 mm, and the depth goes through the sample. The volume to be analyzed was selected perpendicular to the villus–crypt interface (**Figure 1D**). The non-local means filter was used to smooth the inaccuracies caused by noise in the imaging data. The selected volume was segmented to the sample and background with thresholding. The second part was done with in-house Matlab program (MathWorks, Inc., Natick, MA, USA). The surface was extracted from the segmented volume. The Crofton formula was used, and the effective surface area was calculated by dividing the measured area by the theoretical flat area. During the process, the user selects the location of the analysis and the threshold level for the segmentation and everything else is automated.

### Statistics

Patient characteristics are given either as number of cases and percentages or as medians with lower and upper quartiles. Intraobserver and interobserver variations for VH : CrD and surface area were analyzed by the Bland–Altman method, in which the differences between two measurements are plotted against the averages of the two explicit measurements (20, 21). The results are reported as the mean difference between the measurements and limit of agreement, defined as the mean difference ± twice the standard deviation (SD) of the differences. Twice the SD was chosen as the margin of error (21). In the scatterplot, the X-axis shows the mean of the results and the Y-axis the difference between the two intra- or interobserver measurements. Agreement on the measurements was evaluated with the intraclass correlation coefficient (ICC). For better visualization of the measurements, correlation scatterplots are also shown. SPSS Statistics version 27.0.1 (IBM, Armonk, NY, USA) was used for the statistical analyses.

## Results

Altogether, 19 of the participants had CeD, six potential CeD, and 17 were treated CeD in the routine histology, while 16 were non-CeD controls (**Table 1**). Women were overrepresented in all groups, and the median age ranged from 30 to 50 years. All CeD and potential CeD patients presented with HLA DQ2/DQ8, whereas these were lacking from 31.2% of the controls. Similarly, all subjects with untreated or potential CeD, as well as three (18%) of the treated patients, had positive TGA and/or EmA, whereas the controls were invariably seronegative (**Table 1**).

**Table 1:** Clinical and serological findings and celiac disease-associated genetics of the 58 study patients.

	Celiac disease,n = 19	Treated celiac disease, n = 17	Potential celiacdisease^1^, n = 6	Non-celiac controls, n = 16
Age, median (range), years	44 (33-58)	37 (30-56)	50 (44-63)	30 (24-41)
Females, n (%)	17 (89.5)	12 (70.6)	5 (83.3)	12 (75.0)
HLA DQ2/8, n (%)	19 (100)	17 (100)	6 (100)	11 (68.8)
TGA,^2^ median (quartiles), U/L	57.1 (5.8, 101)	2.1 (0.3, 3.0)	6.3 (4.2, 8.0)	0 (0, 0.2)
TGA positive, n (%)	18 (94.7)	2 (11.8)	4 (66.6)	0 (0)
EmA positive, n (%)	17 (89.5)	4 (23.5)	6 (100)	0 (0)

^1^Positive EmA and/or TGA and HLA DQ2/8 with normal duodenal villi in routine histologic evaluation. ^2^Reference <5.0 U/l, highest reported value 101 U/l. EmA, endomysial antibodies; HLA, human leukocyte antigen; TGA, tissue transglutaminase antibodies.

By definition, untreated CeD patients had clearly reduced VH : CrD in the routine histology, whereas the median ratio was at a normal level in treated patients and in those with potential CeD, and even higher among the controls (**Table 2**). The results of the micro-CT measurements reflected these findings, although the median values of other groups except untreated CeD patients were lower (**Table 2**). Of note, four out of the six cases with potential CeD had VH : CrD below 2.0, which is often considered diagnostic for CeD.

**Table 2:** Histological features and micro-CT findings of the 58 study patients.

	Celiac disease, n = 19		Treated celiac disease, n = 17		Potential celiacdisease^1^, n = 6		Non-celiac controls, n = 16	
	Median	Range	Median	Range	Median	Range	Median	Range
VH/CrD, histology	0.2	0.1-1.0	2.6	2.3-3.2	2.6	2.1-3.3	3.3	2.7-3.9
VH/CrD, CT	0.2	0.1-1.1	2.1	1.6-2.8	1.7	1.5-2.8	2.4	1.6-2.8
Surface area^2^, CT	1.4	1.1-2.5	3.9	3.1-4.5	3.5	3.0-3.8	4.7	3.9-5.2

^1^Positive celiac disease serology and HLA DQ2/8 with non-diagnostic histology. ^2^In relation to theoretical completely flat area of 1.0. CT, computed tomography; VH/CrD, villous-height crypt depth ratio.

**Figure 2:**
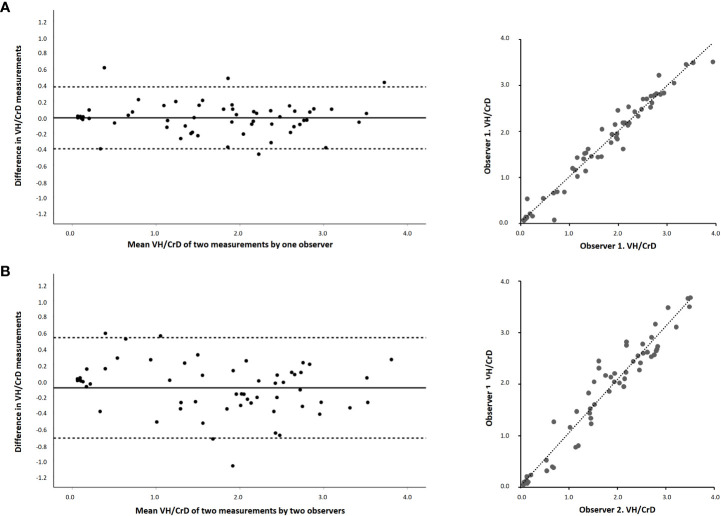
Bland–Altman plots and linear regressions of the morphological measurements of duodenal mucosa with micro-CT imaging by two blinded readers. The panels show villous height crypt depth ratios (VH : CrD) between intraobserver **(A)** and interobserver **(B)** measurements. The x-axis in Bland–Altman shows the mean of the measurements, and the y-axis differences between the measurements. Solid horizontal lines denote the average difference between the readers and dotted lines 95% limits of agreement.

The Bland–Altman analysis for micro-CT morphometry demonstrated a small mean difference for both intra- and interobserver VH : CrD measurements, indicating absence of systematic error (**Figure 2**, **Table 3**). The corresponding limits of agreement were from -0.397 to 0.375 and from -0.719 to 0.537, respectively, and the ICCs were excellent at 0.981 and 0.954, respectively (**Table 3**). The error ranges indicated by twice the standard deviation were 0.397 for intraobserver VH : CrD and 0.536 for interobserver VH : CrD.

**Table 3:** Bland–Altman statistics for micro-CT with absolute values and intraclass correlation coefficients (ICC) for analyzing repeatability and agreement for mucosal morphometry and surface area.

	Mean difference (95% CI)	Standard deviation	Limits of agreement	ICC
VH : CrD				
*Intraobserver*	-0.011 (-0.063 to 0.041)	0.197	-0.397–0.375	0.981
*Interobserver*	-0.080 (-0.175 to 0.007)	0.268	-0.719–0.537	0.954
Surface area				
*Intraobserver*	0.010 (-0.094 to 0.114)	0.395	-0.764–0.785	0.963
*Interobserver*	0.028 (-0.061 to 0.117)	0.342	-0.642–0.698	0.972

CI, confidence interval; VH : CrD, mucosal villus height-crypt depth ratio.

**Figure 3:**
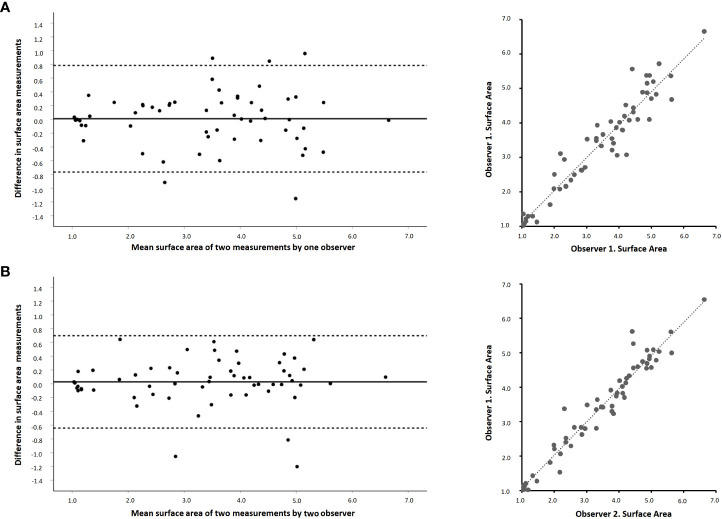
Bland–Altman plots and linear regressions of the surface area measurements of duodenal mucosa with micro-CT imaging by two blinded readers. The panels show the surface area values between intraobserver **(A)** and interobserver **(B)** measurements. The x-axis in Bland–Altman shows the mean of the measurements and y-axis the differences between the measurements. Solid horizontal lines denote the average difference between the readers and dotted lines 95% limits of agreement. The surface area values are given in relation to a theoretical completely flat area with a value of 1.0.

The mean differences for the intra- and interobserver micro-CT measurements of the mucosal surface area were even lower than those of VH : CrD, again suggesting a negligible systematic error (**Figure 3**, **Table 3**). The limits of agreement were from -0.764 to 0.785 for the intraobserver analyses and from -0.642 to 0.698 for the interobserver analyses, and ICCs 0.963 and 0.972, respectively (**Table 3**).

## Discussion

Both intraobserver and interobserver reproducibility for the morphometric measurements with micro-CT proved excellent with practically no systematic error between readers. For comparison, the most widely used grouped classifications in conventional histology, although fairly good for distinguishing between severe atrophy and healthy mucosa, have demonstrated wide intra- and interobserver variation for the less advanced lesions commonly seen in CeD patients (1, 13, 22–24). Ideally, measurement of quantitative VH : CrD can provide more accurate and reproducible results, particularly when special emphasis is placed on correct orientation of the biopsy cuttings (**Figure 1B**) (7, 9, 13, 23). However, acquiring acceptable sections for histomorphometry requires skilled personnel and is laborious and time-consuming (8, 9, 13), making it impractical for clinical routine. In fact, achieving an appropriate cutting angle may not be possible even with rigorous effort due frequently to tissue availability. By contrast, micro-CT allows fast, accurate, and reproducible digital cutting and morphometric measurement with optimal angles (**Figure 1C**).

As a novel approach, surface area measurements with micro-CT yielded similarly excellent accuracy and reproducibility as the morphometry. An additional reason for the good reproducibility is likely the partial automatization of the reading process, enabled by the real 3D image set, which may be further extended in the future (25). A well-known concern in CeD is patchiness of the mucosal lesion between or even within the biopsies, this possibly leading to misdiagnosis and to reduced accuracy in the aforesaid longitudinal trials (6, 8, 13). Visual inspection of the 3D reconstructions enables selecting an intact measurement site for the surface area, and, simultaneously, measurement of a higher number of villi compared to the cuttings reduces random variation (**Figure 1D**). Of note, the villous surface areas have previously also been measured indirectly from traditional biopsy samples, but with a much less sophisticated and practical approach (26). We believe that the surface area measurement with micro-CT could provide major benefits in the future pharmaceutical studies in CeD and with diagnostically challenging cases.

Excluding untreated CeD, micro-CT produced lower median VH : CrDs than the conventional histology. It is possible that the reader selects an area with the most evenly distributed and that the shortest villi form the 3D reconstruction, whereas in routine histology the most “representative” cutting with longer villi is preferred. It should be borne in mind that the exact morphometric cutoff for the CeD diagnosis remains debatable even with traditional histology (14, 24, 27–29). The villous length may also vary depending on the anatomical location within the intestine (29), emphasizing the use of standardized sampling sites in longitudinal studies. As regards the surface area, here the lowest factor among the non-CeD controls was 3.9, but additional studies specifically addressing the diagnostic value are needed. Notably, four subjects with potential CeD already had diagnostic VH : CrD with micro-CT imaging, and some treated patients showed values indicating incompletely mucosal recovery, a condition known to be common even in case of strict dietary adherence (30). Although the same cutoffs may not apply directly, this nevertheless suggests a superior sensitivity of micro-CT for borderline mucosal lesions compared with the conventional histology. Further studies on this issue and optimal VH : CrD cutoffs are however called for.

There are several ongoing studies testing pharmaceutical therapies for CeD (31). Taavela et al. demonstrated improved accuracy for quantitative VH : CrD compared with grouped classification to detect minor mucosal changes during prospective intervention, with a change of ≥0.4 being considered significant according to the margins of error (9, 13). Our results are in line with this, which was to be expected as the same morphometric outcome was used, but with micro-CT, this was accomplished with much less effort. Given the abovementioned reduced random variation and the exponential nature of surface area compared with morphometry, it could be expected to be an even more accurate and sensitive method. The margin of error (2SD) for the surface area measurement was approximately 0.7–0.8, which should be confirmed in future clinical studies.

Our main strengths were the well-defined cohort of CeD and non-CeD individuals, representing a wide range of histological damage, and the use of previously optimized procedures for the imaging. As a limitation, the study sample size was only moderate, although we considered it sufficient for the conducted validation analyses. Furthermore, micro-CT also has some technical limitations that should be addressed. First, the resolution—although adequate for the morphometric analyses—is lower than that with histology. Second, we did not quantify the degree of mucosal inflammation at this point, but this should be possible in the future utilizing specific contrast agents (30). In fact, the mucosal cell count is less sensitive for reading errors even with conventional histology (32, 33). Finally, the equipment and expertise needed for micro-CT is available only in specialized centers, which may increase costs and limit the wide-scale use of the methodology, but it should be quite straightforward to ship the biopsies for centralized imaging.

## Conclusion

Measurement of small-bowel mucosal morphology and surface area using digitalized 3D micro-CT reconstructions is accurate and reproducible with excellent intraobserver and interobserver agreements. The novel technology provides a robust tool for assessing diagnostically ambiguous cases in CeD and for academic and pharmaceutical trials. Future research could explore the performance of micro-CT particularly in diagnostically challenging situations such as potential CeD and for the diagnosis of other intestinal diseases involving morphological lesions.

## Data availability statement

The data that support the findings of this study are available on request from the corresponding author. The data are not publicly available due to privacy and ethical restrictions. Requests to access the datasets should be directed to kalle.kurppa@tuni.fi.

## Ethics statement

The studies involving human participants were reviewed and approved by The Regional Ethics Committee of Pirkanmaa Hospital District. The patients/participants provided their written informed consent to participate in this study.

## Author contributions

All the authors listed met the authorship criteria. JV, KKu, MH, JT, and HH designed the study and contributed to the data analysis and interpretation and drafting of the manuscript. KL, IT, KKa, PS, and JH critically reviewed the analysis and made a substantial additional contribution to the manuscript. All authors reviewed the manuscript and approved the final version.

## Funding

The work was funded by the Academy of Finland, the Finnish Funding Agency for Technology and Innovation, the Sigrid Juselius Foundation, the Foundation for Pediatric Research, the Päivikki and Sakari Sohlberg Foundation, the University Consortium of Seinäjoki, and the Competitive State Research Financing of the Expert Area of Tampere University Hospital. The funders had no role in the design or conduct of the study.

## Conflict of interest

The authors declare that the research was conducted in the absence of any commercial or financial relationships that could be construed as a potential conflict of interest.

## Publisher’s note

All claims expressed in this article are solely those of the authors and do not necessarily represent those of their affiliated organizations, or those of the publisher, the editors and the reviewers. Any product that may be evaluated in this article, or claim that may be made by its manufacturer, is not guaranteed or endorsed by the publisher.
